# Stigma and General Help-Seeking Behaviour Among Rural Residents at Al-Karad Village, Ad Dabbah Northern State, Sudan 2022

**DOI:** 10.1192/bjo.2025.10141

**Published:** 2025-06-20

**Authors:** Danya Ibrahim, Mohja Mohamed, Lana Mustafa, Marwa Abdelwhab Alameen, Emtenan Khurasani Ahmed

**Affiliations:** Khartoum University, Faculty of Medicine, Khartoum, Sudan

## Abstract

**Aims:** This study aimed to assess help-seeking intentions for personal and emotional problems, suicidal thoughts, and sources of help, and to evaluate perceived stigma about psychological treatment among participants.

**Methods:** This cross-sectional study was conducted in December 2022. A total of 101 participants filled out an online survey through interviews. The questionnaire is composed of sociodemographic characteristics; 14 items of a structured standardized questionnaire of general help-seeking behaviour using the General Help-Seeking Questionnaire (GHSQ) and Stigma scale for receiving psychological help (SSRPH).

**Results:** The majority of the participants were above 30 years (70.3%). The level of stigma was predominantly low (58%) and high stigma was at 24%. The majority of the participants experienced suicidal thoughts (68%) and mostly were males. Most of the participants preferred to seek help from a mental health professional (M=5, SE=0.26) followed by their partner (M=4.5, SE=0.26) then parents and family members (M=4.4, SE=0.25).

**Conclusion:** Stigma has a significant impact on the people of Sudan, affecting individual behaviours and creating barriers for others. Recommendations include focusing on mental health advocacy, reaching out to individuals with suicidal thoughts, and providing mental health services in rural areas, particularly in Al-Karad village.

